# Increments and Duplication Events of Enzymes and Transcription Factors Influence Metabolic and Regulatory Diversity in Prokaryotes

**DOI:** 10.1371/journal.pone.0069707

**Published:** 2013-07-29

**Authors:** Mario Alberto Martínez-Núñez, Augusto Cesar Poot-Hernandez, Katya Rodríguez-Vázquez, Ernesto Perez-Rueda

**Affiliations:** 1 Departamento de Ingeniería de Sistemas Computacionales y Automatización, Instituto de Investigaciones en Matemáticas Aplicadas y en Sistemas, Universidad Nacional Autónoma de México, Ciudad Universitaria, México D.F., México; 2 Departamento de Ingeniería Celular y Biocatálisis, Instituto de Biotecnología, Universidad Nacional Autónoma de México, Cuernavaca, Morelos, México; American University in Cairo, Egypt

## Abstract

In this work, the content of enzymes and DNA-binding transcription factors (TFs) in 794 non-redundant prokaryotic genomes was evaluated. The identification of enzymes was based on annotations deposited in the KEGG database as well as in databases of functional domains (COG and PFAM) and structural domains (Superfamily). For identifications of the TFs, hidden Markov profiles were constructed based on well-known transcriptional regulatory families. From these analyses, we obtained diverse and interesting results, such as the negative rate of incremental changes in the number of detected enzymes with respect to the genome size. On the contrary, for TFs the rate incremented as the complexity of genome increased. This inverse related performance shapes the diversity of metabolic and regulatory networks and impacts the availability of enzymes and TFs. Furthermore, the intersection of the derivatives between enzymes and TFs was identified at 9,659 genes, after this point, the regulatory complexity grows faster than metabolic complexity. In addition, TFs have a low number of duplications, in contrast to the apparent high number of duplications associated with enzymes. Despite the greater number of duplicated enzymes versus TFs, the increment by which duplicates appear is higher in TFs. A lower proportion of enzymes among archaeal genomes (22%) than in the bacterial ones (27%) was also found. This low proportion might be compensated by the interconnection between the metabolic pathways in Archaea. A similar proportion was also found for the archaeal TFs, for which the formation of regulatory complexes has been proposed. Finally, an enrichment of multifunctional enzymes in Bacteria, as a mechanism of ecological adaptation, was detected.

## Introduction

An important clue to understanding how prokaryotes have evolved and how they exist in their modern forms can be addressed by comparing their transcriptional and enzymatic repertoires. The availability of more than 3,000 organisms for which complete genome sequences have been determined, belonging to the three cellular domains of life, provides the opportunity to conduct evolutionary and functional studies. In this respect, the diversity of lineages, genome sizes, and lifestyles are widely represented in the universe of organisms for which genomic information is available, such as the archaeon *Nanoarchaeum equitans*, an obligate symbiont with one of the smaller genomes, 0.491 Mbp [1]–[3], or the largest bacterial genome sequenced so far (10 Mbp), the filamentous nitrogen-fixing cyanobacterium *Nostoc punctiforme* [4]–[9]. Consequently, assessing the distribution and content of genes associated with enzymatic reactions and gene regulation in sequenced genomes, the evolution of metabolic and regulatory capabilities of prokaryotic cells can be inferred.

Under this perspective, in this work, an exhaustive analysis of enzymes and DNA-binding transcription factors (TFs) was conducted to evaluate their abundance and distribution across 794 non-redundant genomes from *Bacteria* and *Archaea* cellular domains. From this study, we found that enzymes and TFs follow power-law behavior and that archaeal organisms exhibit a smaller repertoire of enzymatic and regulatory proteins than bacterial organisms. The rate of change (derivative) associated with the power-law behavior exhibits a negative trend for enzymes, while for TFs is positive. These findings suggest that the number of acquired enzymes diminishes as genome size increases; on the contrary, the number of TFs acquired increases as the size of the genome increases. This inverse behavior between the rates of incremental changes in the numbers of TFs and enzymes can be explained in terms of decreasing enzymatic diversity of prokaryotes due to a re-use of existing enzymes (referred as “tools”) already encoded in the genome. Thus, as the genome of an organism becomes larger, it acquires a smaller amount of new tools to perform a new metabolic task because the larger “toolbox” is more likely to contain the necessary enzymes for the new function [10]. While an expansion of the regulatory capability would optimize matter acquisition and processing energy through an increase in the number of TFs acting on metabolic enzymes. In addition, TFs exhibit a low number of duplications, in contrast with the high number of duplications associated with enzymes. Despite the greater number of redundant enzymatic sequences, the rate of increments for duplicates is higher for TFs than for enzymes. Structural domain contents, functional promiscuity, and enzyme pathway relationships were also investigated. From these data, it was determined that the low proportion of enzymatic proteins associated with archaeal genomes can also be partially explained by the low number of metabolic pathways identified in those organisms and by the functional plasticity associated with their proteins that participate in diverse metabolic pathways. Finally, we suggest that one of the strategies by which prokaryotic organisms contend with changing environments is by increasing the number of multifunctional enzymes, which can confer to the cell the ability for adaptation to different ecological niches.

## Results

### Enzymes Follow a Power-law Behavior in Relation to Genome Size

**Figure 1:**
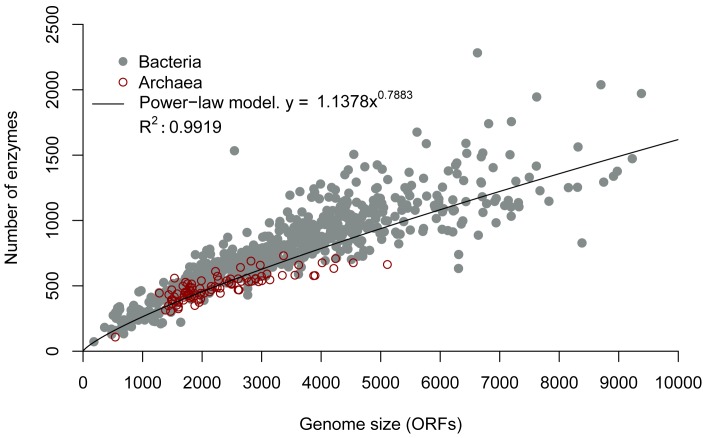
Abundance of enzymes as a function of genome size. On the *x* axis, genomes are sorted by the number of ORFs. The abundance of enzymes for each genome is shown on the y axis. Each dot corresponds to one genome. *Bacteria* and *Archaea* genomes are indicated by gray and filled or red and empty dots, respectively. The power-law function (black line) and R^2^ adjustment are also indicated *y* = 1.1378*x* ^0.7883^.

In order to assess the abundance of the enzymatic repertoires in *Bacteria* and *Archaea* genomes, 794 non-redundant organisms were analyzed by using diverse bioinformatics tools and database assignments. In this work, enzymes were selected on the basis of their E.C. numbers, PFAM, COG and Superfamily annotations in addition to the annotations deposited in the Kyoto Encyclopedia of Genes and Genomes (KEGG) database. Once the enzyme dataset was selected, the correlation between the number of detected enzymes and genome sizes (open reading frames [ORFs]), based on calculation of Spearman’s rank correlation coefficient, was analyzed. From this analysis, the occurrence of enzymes correlated with the genome size, with a strong Spearman’s coefficient of 0.93 (*p*-value <2.2e^−16^). To determine whether there is a linear function between the distribution of enzymes and genome size, a standard residual plot analysis was performed. The residual analysis showed that the distribution of the enzymes with respect to genome size was not linear but followed a power-law behavior, with a correlation coefficient (*R^2^)* of 0.99 between data and the power-law fitting function (Figure 1). The exponent in the power-law function of our model was 0.78, which is within the range of exponents of protein families with functions related to metabolism or transport, as well as DNA replication and repair, as previously reported [11]–[14]. To reinforce the notion of the power-law behavior previously described, a sliding-window boxplot analysis was accomplished by using five different sets of windows (11, 22, 33, 44, and 55) with different length sizes (836, 418, 278, 209, and 167 ORFs). From this analysis, exponents in the power-law ranging from 0.74 to 0.80 were obtained, being consistent with the complete data set, suggesting that our analysis is robust (Figure S1 and Table S1). When the power-law fitting function was calculated using the enzymatic repertoires in *Bacteria* and *Archaea* as independent data sets, similar results were found (data not shown). To discard the possibility of overrepresentation of sequenced bacterial genomes in the observed results or an uneven sampling of genomes with different size-ranges, we analyzed the abundance and distributions of enzymes in equivalent sets of *Bacteria* and *Archaea*, i.e., 89 bacterial genomes were selected with similar genome sizes to the archaeal genomes considered in this study (referred to here as the “bacterial subset”). In table 1 we show the properties of the bacterial subset and the archaeal genomes. From this analysis, results similar to those for the complete data set were obtained, reinforcing the notion of a power-law behavior of enzymatic capabilities of prokaryotic genomes, in contrast to the linear model previously proposed [13].

**Table 1:** Comparative analysis of enzymes of *Archaea* and *Bacteria* genomes.

Parameter	*Bacteria*	Bacterial subset#	*Archaea*
Total of available sequenced genomes	705	89	89
Average of ORFs	3,314	2,280	2,292
Average of enzymes	764	598	487
Proportion of enzymes/ORFs*	25.15	27.01	22.38
Average of unique metabolic pathways per organism	71	66	58
Number of different superfamily domains	364	336	287
Number of different superfamily domains/total of superfamily domains*	32.28	36.18	38.9
Average of mutifunctional enzymes	68	55	41
Number of multifunctional enzymes/total of enzymes*	8.8	9.2	8.3
Spearman’s rank coefficient	0.93 (*p*-value <2.2e^−16^)	0.84 (*p*-value <2.2e^−16^)	0.82 (*p*-value <2.2e^−16^)

Values are averages for each group presented.

* : Expressed as a percentage; Spearman’s rank correlation coefficient between enzymatic sequences and genome size. The superfamily domains were obtained from Superfamily database [18].

# : Bacterial subset contains 89 genomes with similar sizes (ORFs) as their archaeal counterpart.

### *Archaea* organisms contain a smaller repertoire of enzymatic proteins and metabolic pathways than *Bacteria*

In order to compare the repertoire of enzymatic proteins in all the prokaryotic genomes, the content of this class of proteins was exhaustively evaluated. From this analysis, it was determined that bacterial and archaeal genomes exhibit a different average number of enzymes, 764 and 487, respectively (Wilcoxon test, *p*-value <2.2e^−16^) (Table 1). Hence, 1.5 times more enzymatic sequences in bacterial than in archaeal genomes were detected. When the proportion of enzymes was obtained in relation to the genome size, according to the ratio of enzymatic sequences versus total ORFs per genome, around one-quarter of the bacterial genes encode for enzymes (25.1%), whereas in *Archaea* this proportion is approximately one-fifth of the genome (22.3%) (Table 1). A similar result was found when a histogram of the fraction of metabolic enzymes comparing *Archaea* and *Bacteria* within a bin of roughly equal genome sizes was achieved (Figure S2, top). To exclude a possible bias as a consequence of the number of bacterial genomes, the bacterial subset was evaluated, identifying an average of 598 enzymes, versus 487 in archaea genomes, being statistically significant (Wilcoxon test, *p*-value = 2.44e^−06^). The proportion of the enzymatic content showed an increase to almost one-third for the bacterial subset (27%), which was statistically different from the value previously obtained for the archaeal genomes (Wilcoxon test, *p*-value = 8.486e^−06^) (Table 1). These findings not only suggest more diversified metabolic capabilities in *Bacteria*, which can carry out cellular processes from a wide range of metabolizable substrates and can be found in ecological niches where the variables may show high fluctuations, but also that archaeal metabolism might be constrained to particular environmental conditions, where variables such as temperature, salt concentration, and pH can be extreme and metabolizable substrates are limited. For example, the methanogenic *Archaea*, for which the number of substrates used to carry out methanogenesis may be restricted to CO_2_/H_2_, formate, and/or acetate [15] and archaeal organisms with an only hydrogen-based energy metabolism in more stable, although extreme, environments [16].

In addition, we evaluated whether the low enzyme content observed in *Archaea* is related to the small number of metabolic pathways. To determine the number of metabolic pathways in all the genomes, an account of unique metabolic pathways annotated within the KEGG database for each organism was obtained. From this analysis, the average numbers of metabolic pathways detected were 66 for the bacterial subset and 58 for the archaeal genomes (Wilcoxon test, *p*-value = 2.252e^−08^) (Table 1), suggesting a lower proportion of metabolic pathways in *Archaea* than in *Bacteria*. It is interesting to note that the carbohydrate, amino acids and cofactors, and vitamins metabolisms are the most prevalent in all the bacterial and archaeal genomes. On the contrary, biosynthesis of other secondary metabolites, glycan biosynthesis, and nucleotide metabolism are underrepresented in the organisms analyzed here (Figure S2, bottom). Another important question asked was whether there was an association between all the enzymes and their corresponding metabolic pathways, i.e., the proportion of shared enzymes in more than one pathway. In this address, enzymes associated with three pathways were identified in higher proportions in *Bacteria* than in *Archaea*, and the opposite was observed for enzymes that participate in two or six pathways, i.e., such enzymes were more frequent in *Archaea* than in *Bacteria* (Figure S3). Thus, the low enzymatic repertoire identified in *Archaea* could be associated to the low number of metabolic pathways reported in the databases for these organisms, where the apparent lack of enzymes could be compensated by the increment in the proportion of enzymes participating in two or six pathways, increasing the betweenness in this cellular domain. In this context, it has been reported that metabolic pathways present in *Archaea* have a high betweenness, with more central pathways located at the cross-points of many pathway pair communications [17], reinforcing our previous observations.

**Figure 2:**
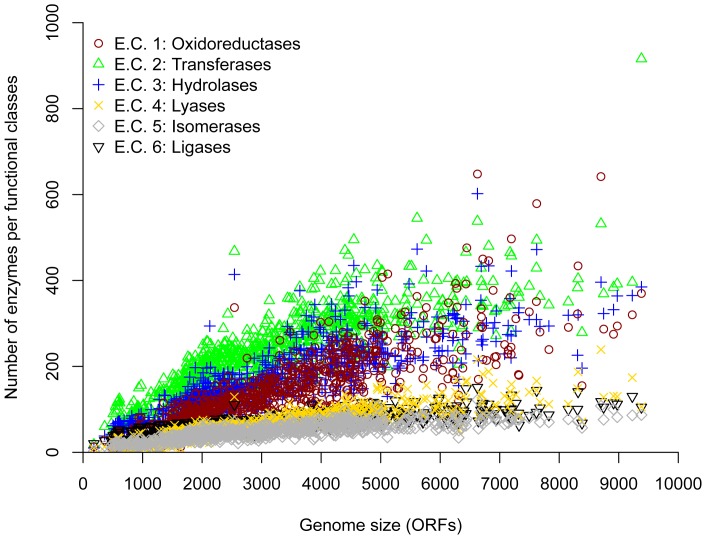
Abundance of different E.C. classes in Bacteria and Archaea genomes. On the *x* axis, genomes are sorted by the number of ORFs. The abundance of the six E.C. classes is shown in *y* axis.

To elucidate the individual contribution of the broad enzymatic classes (E.C. assignments) to the power-law behavior previously described, we analyzed their abundance in all the genomes. From this analysis, it was evident that the three E.C. classes most represented in all the genomes corresponded to transferases (E.C. 2), hydrolases (E.C. 3), and oxidoreductases (E.C. 1). Indeed, these three classes contributed significantly to the distribution trend previously described. In contrast, lyases (E.C. 4), ligases (E.C. 6), and isomerases (E.C. 5) were less abundant (Figure 2). In archaeal genomes, the oxidoreductases are the second most abundant group of enzymes, unlike bacterial genomes, in which the second most abundant class corresponds to the hydrolases (Figure S4). The increase of enzymes associated with redox processes in archaeal genomes reflects the footprint of inhabited environments associated with these organisms, such as anoxic environments, for which the organisms have developed the ability to use hydrogen-based energy metabolism [16]. In this regard, the electron transfer process for energy use (consumption and generation) associated with oxidoreductases and its metabolite electron carriers, NAD(P)(H) and FAD(H), are favored in archaeal organisms as a response to the ecological environments they inhabit, in which energy and matter use are optimized.

### Structural Domains are More Diverse for Archaeal than Bacterial Enzymes

To gain insights into the diversity of enzymes detected in bacterial and archaeal genomes, the structural domains associated with the enzymatic repertoire were evaluated using the superfamily domains assignments of Superfamily database [18]. The proportion of different superfamily domains in relation to the total superfamily domains detected per genome was calculated. From this analysis it was found that in average the fraction represented in the bacterial subset was 36%, whereas in *Archaea* was 39% (Wilcoxon test, *p*-value = 5.004e^−05^). Table 1. Therefore, a lower proportion of different structural domains was identified in *Bacteria* than in *Archaea*. The higher proportion of different structural domains in *Archaea* may be an associated mechanism to compensate for the relatively low abundance of identified enzymes, allowing greater participation of archaeal enzymes in a higher number of metabolic pathways, as described below, which may contribute to the betweenness of metabolic networks [17].

### Enzyme Promiscuity, a Fitness Mechanism of an Organism to Adapt to its Ecological Niche, is Higher in *Bacteria* than in *Archaea*

Considering the differences between the numbers of enzymatic proteins and metabolic pathways, in *Bacteria* and *Archaea* previously described, we decided to evaluate whether an increase in enzymes with multiple functions, i.e., promiscuous enzymes that exhibit more than one different E.C. number, compensates for the low number of enzymes in *Archaea*. Multifunctional enzymes have been considered a mean for evolution of metabolic pathways. From multifunctional enzymes that catalyze consecutive steps, the pathways might have evolved by duplication and diversification of these precursors to form specific and efficient enzymes that catalyze only one step in the new pathway [19], [20]. Based on this approach, the number of promiscuous enzymes identified was higher, on average, in the bacterial subset (55) than in *Archaea* (41) (Wilcoxon test *p*-value = 0.0023) (Table 1). When the fraction that represented the promiscuous enzyme sequences in relation to all enzymes by organism was evaluated, a significant difference in the ratios for the bacterial subset (9.15%) versus *Archaea* (8.3%) was found (Wilcoxon test, *p*-value = 0.034). The number of promiscuous enzymes in the bacterial subset preserved the difference observed in the analysis of all bacterial genomes, i.e., there was a greater number of promiscuous enzymes in bacterial than in archaeal organisms. A greater number of promiscuous enzymes in *Bacteria* could compensate for the fact that these organisms have a lower percentage of different structural domains than *Archaea*, as previously discussed. In this regard, *Bacteria* can perform a great number of enzymatic reactions without the need to change an entire domain, by modifying specific positions that change the catalytic activity or the substrate recognition of the enzyme. Mutation of specific positions in the metabolic genes can lead to the development of new catalytic activities in the encoded enzymes, which is a fast evolutionary mechanism in comparison to modifications in the entire domain; thus, Bacteria can respond more quickly to fluctuations in variables at their ecological niches. An example of duplication and divergence of enzymes that conserve the same domain is observed in the biosynthesis of tryptophan and folate molecules in *Escherichia coli.* The pathways involved in the first step of the biosynthesis of tryptophan and folate start off with the same substrate, chorismate, but the final product is completely different. The *trpE* and *trpG* genes encode proteins involved in the conversion of chorismate to anthranilate in the tryptophan pathway, while the products of the *pabA* and *pabB* genes, paralogues of *trpE* and *trpG*, carry out the conversion of chorismate to *p*-aminobenzoate [21]–[23].

In addition, it has been observed that generalist enzymatic reactions (i.e., of promiscuous enzymes) have low metabolic fluxes, while specialist enzyme reactions (i.e., of specific enzymes) maintain high metabolic fluxes, as estimated for the steady-state metabolic flux rates for *E. coli* enzymes [24]. Similarly, when the regulatory mechanisms, which act on generalist and specialist enzymes, were quantified, less regulation in the generalist enzymes was found, while allosteric, uncompetitive, and noncompetitive regulatory interactions were enriched among specialists [24]. These data complement our observation of the importance of promiscuous enzymes in bacteria as an evolutionary mechanism of fitness to different ecological niches, from the metabolic perspective. The presence of more promiscuous enzymes allows for internal metabolic fluxes that can vary according to environmental fluctuations; therefore, there is less regulation, which allows faster reprogramming of gene expression. In contrast, specialist enzymes, which are found in greater numbers in *Archaea*, maintain high metabolic fluxes with a higher sensitivity to environmental changes and are identified in more stable and even extreme environments.

### TF Distributions Follow a Similar Trend as Enzymatic Proteins

**Figure 3:**
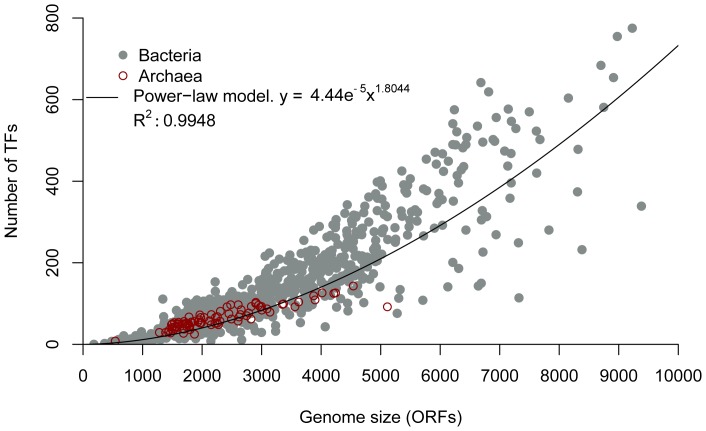
Abundance of TFs as a function of genome size. On the *x* axis, genomes are sorted by the number of ORFs. The abundance of TFs for each genome is shown on the y axis. The power-law equation (black line) and R^2^ adjustment are also indicated. *y* = 4.44e-05*x* ^1.8044^. Gray filled points represent *Bacteria*; red empty points represent *Archaea*.

In this section, we describe how the number and diversity of DNA-binding TFs can also influence cellular complexity. Bacterial and archaeal genomes were analyzed in a similar fashion as for the enzymatic repertoires, i.e., numbers of TFs were evaluated as a function of genome size. From this analysis, the Spearman’s rank correlation coefficient was 0.91 (*p*-value <2.2e^−16^), showing a strong positive correlation between TFs and genome size (measured by ORFs number). Residual analysis showed that the distribution of TFs detected with respect to genome size was not linear, and the function that best fitted the distribution was the power-law (*R^2^* = 0.99) (Figure 3). Similarly, the power-law fitting function exponent (1.80) was within the range reported in other studies for protein families classified as regulators [12], [13]. When a sliding-window boxplot analysis was achieved, the consistence of the power-law fitted function was confirmed (Figure S5).

Therefore, TFs increase in their number as the size of the genome increases. Although in small genomes the number of TFs is lower than in larger genomes, mainly in those described as intracellular or opportunistic pathogens [25], the higher number of TFs in larger genomes does not necessarily imply diversity of families, but instead an increase in the size of some families of TFs, as it has been previously described [26], [27]. This increase in the number of any given family seems to stem primarily from lineage-specific proliferation of families of paralogous genes [12], [13]. In addition, recent results suggest that a few regulatory elements identified in small genomes could compensate for the regulation of the entire genome, with an increase in the number of DNA-binding sites per element, in contrast to the high number of elements identified in large genomes, which control a smaller proportion of DNA-binding sites, on average [28].

### TF Content and Domain Diversity

In this section we compare the repertoires of regulatory proteins between *Bacteria* and *Archaea* in terms of their TF contents and structural diversity (superfamily domains). From this analysis, it was found that the bacterial and archaeal genomes exhibited a different average number of TFs, 150 and 73, respectively (Wilcoxon test, *p*-value = 2.11e^−8^) (Table 2). When the proportion of TFs was analyzed with respect to the total genome, almost 4% of genes encoded TFs in bacterial genomes, while in archaeal genomes around 3% of the gene products were TFs. When the analysis was performed with the bacterial subset, neither the number nor the proportion of TFs showed a significant difference (Wilcoxon test, *p*-value >0.05) (Table 2). To determine the diversity of structural domains in TFs, the identification of domains annotated within the Superfamily database was carried out. The results showed that the number of different superfamily domains in bacterial TFs is greater than in archaeal regulators, with average numbers of 28 and 20, respectively. However, when the proportion of different superfamily domains associated to TFs was calculated in relation to all superfamily domains present in each genome, a higher proportion of different superfamily domains in the archaeal genomes was found, with an average of 28% different domains, versus Bacteria, with an average of 24% (Wilcoxon test, *p*-value = 1.39e^−10^) (Table 2). When the structural domains analysis was performed with the bacterial subset, the proportion of different superfamily domains did not differ substantially from the *Archaea* proportion. From the data obtained in our analysis of the content and diversity of superfamily domains in TFs, a bias was observed, probably as consequence of the smaller number of sequenced archaeal genomes. However, it has been reported an apparent deficit of TFs in archaeal genomes with similar sizes to the bacterial genomes, such as those of *Methanosarcina acetivorans* and *Haloarcula marismortui* with a similar sizes to the bacteria *E. coli* K12 but with a lower proportion of TFs [15], which agrees with our data analysis using the full set of bacterial genomes. One possibility for the apparent lack of proteins dedicated to regulation of gene expression in *Archaea* is that these proteins form various multimeric complexes, as happens in eukaryotes and, depending on which complex is formed, the target sites in the DNA will be different [15]. This possibility of forming complex regulatory structures may be favored by the higher proportion of diversity of structural domains present in *Archaea*, as found in our study and mentioned above, as such diversity would increase the number of different combinations and would avoid repetition of the elements that make up the complex.

**Table 2:** Comparative analysis of TFs of *Bacteria* and *Archaea*.

Parameter	*Bacteria*	Bacterial subset#	*Archaea*
Number of TFs	150	81	73
Number of TFs/ORFs*	3.9	3.31	3.16
Number of different superfamily domains	28	23	20
Number of different superfamily domains/total of superfamily domains*	24.88	29.27	28.15

Values are averages for each group presented.

* : Expressed as a percentage. The superfamily domains were taken from Superfamily database [18].

# : Bacterial subset contains 89 genomes with similar sizes (ORFs) as their archaeal counterpart.

### Rates of the Incremental Changes in Enzymes Numbers and TFs are Different in Prokaryotic Genomes

**Figure 4:**
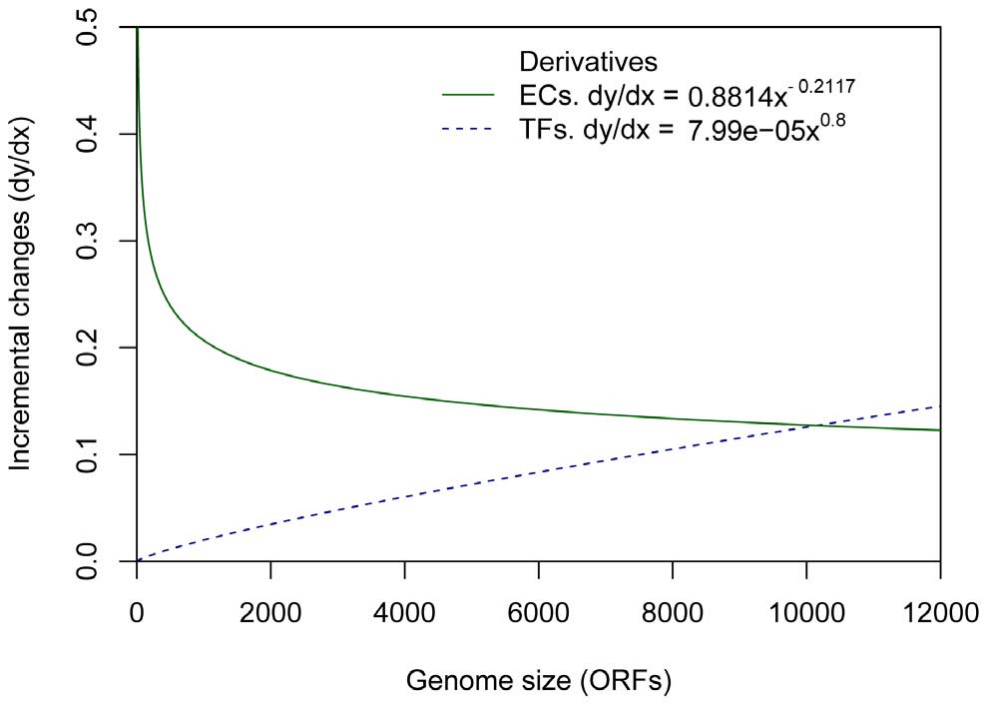
Derivatives of enzymes and TFs abundances. The increments of enzymes and TFs ORFs (*y* axis) were estimated from the derivatives of metabolic (solid green line) and regulatory (blue dashed line) functions, calculated as a function of the genome size (*x* axis).

Based on the previous results, the abundance of enzymes and TFs increase according to genome size. These increases in metabolic and regulatory contents of the genomes seem to be coupled, so that an increase in the number of metabolic enzymes in a genome is usually accompanied by additional new TFs regulating these enzymes [27]. In order to study the coupling between the metabolic and regulatory contents, the rates of incremental changes in the numbers of enzymes and TFs with respect to the increase in genome complexity were analyzed, based on the calculation of the derivative of the power-law fitting function adjusted to the abundance of enzymes and TFs previously described. From these data, it was identified that the rate of incremental changes in metabolic enzymes diminishes as the genome size grows with an abrupt drop in genomes with less than 1,500 ORFs (Figure 4). Above this level, the rate follows a slight decay with a gentle slope. In contrast, the rate of incremental changes of TFs increases as a function of the genome size (Figure 4). The acquisition rate for TFs identified here is lower than that previously reported by Ranea et al. [13] for families of regulatory proteins. This variation could be due to the data sets from which the models were obtained, as our set of regulatory sequences was minor due to the specificity in the identification of TFs, while Ranea et al. [13] employed superfamilies for counting, i.e., we considered TFs as those proteins that activate or repress gene expression but do not belong to the transcriptional basal machinery, and in consequence sigma factors, antiterminators, terminators, and sensor proteins, among other proteins were excluded from the resulting data set.

In this context, we found that the maximum rate of incremental changes for enzymes corresponded to genomes with less than 2,000 genes, while genomes above that size acquired enzymes at a slower speed. Maslov et al. [10], propose that the number of added enzymes (“tools”) systematically decreases with the proportion to which the organism has already explored the universe of available metabolic reactions and thus, indirectly, with the size of its genome, which agrees with the decrease in the rate of incremental changes in numbers of enzymes found in this study. Several approaches to estimate the upper bound of the gene number have been proposed, such as the viewpoint of “microeconomic principles” where the acquisition of enzymes is set equal to the “bacterial revenue” and the acquisition of TFs with a “logistical cost”, and then finding the point of maximization between the metabolic complexity (“revenue”) and the number of regulators (“logistical cost”) [13]. Based on a similar approach, the intersection between the derivatives for enzymes and TFs was identified at 9,659 genes. At this point, the metabolic gain is equal to expenses for the increase in regulation; after this point, the regulatory complexity grows faster than metabolic complexity every time genome complexity increases. Although the average fraction of the genome that encoded enzymes (25%) was greater than for TFs (4%); when analyzing the proportion of the genome that encodes enzymes and TFs with respect to genome sizes, a decrease in the enzyme fraction and an increase in the TF fraction were found, consistent with acquisition rate previously described for enzymes and TFs. Analysis of the fraction of the genome that represented enzymes in three different classes of genome size (<3,000, between 3,000 and 6,000, and >6,000 genes), shows a decrease in enzyme content when moving from genomes containing fewer than 3,000 genes to those larger than 6,000 genes. Conversely, the fraction of TFs per genome increased from small genomes (below 3,000 genes) to larger genomes (greater than 6,000 genes). The differences between the fractions of enzymatic proteins and between TFs fractions with respect to the genome sizes showed a statistically significant difference (Wilcoxon test, *p*-value = 2.752e^−12^). This finding agrees with that reported previously by Cases, et al [25], whose study showed that larger genomes harbor more TFs per gene than small ones while there is a greater overrepresentation of small-molecule metabolism enzymes per gene in smaller genomes than in large genomes. This inverse behavior between rate of TFs and enzymes increments can be explained within the context of cell capacity compensation, where a decrease in the rate of acquisition of new enzymes of prokaryotes in relation to an increase in genome size is compensated by an expansion in the regulatory ability to optimize matter acquisition and processing of energy.

### Increment Rates of Duplicated Sequences Differ between Enzymes and TFs in Prokaryotes

**Figure 5:**
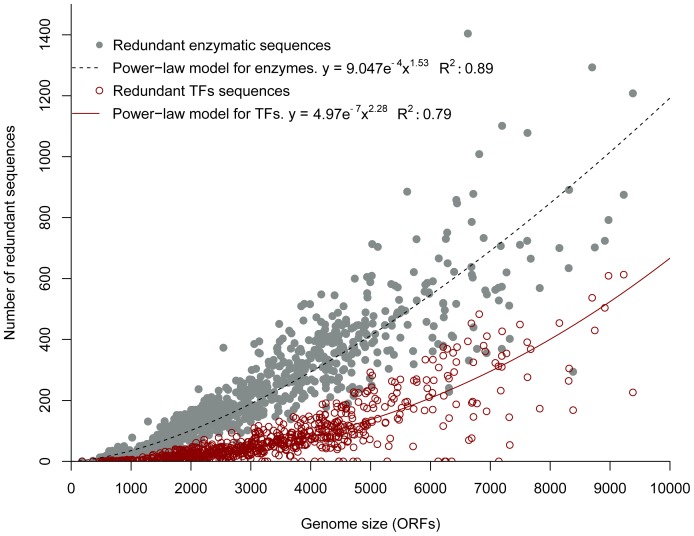
Abundance of duplicated enzymes and TFs as a function of genome size. The total numbers of all duplicated enzymes and TFs were calculated for each species (*y* axis) and plotted against the sizes of the 794 species, in ORFs (*x* axis). The power-law equation and R^2^ adjustment are also indicated. The power-law fitting function is shown by the black dashed line (enzymes) and the red solid line (TFs). Gray filled points represent enzymes; red empty points represent TFs.

Gene duplication has been described as a source of raw material for the generation of new functions in prokaryotes. In models designed to explain the change in the sizes of protein families with respect to the sizes of their genome, duplication processes have been reported to play an important role. To evaluate the effects of duplication processes in the repertoire of enzymes and TFs in all the genomes, we applied a Blastp “all-against-all” search sequence, as described below in Material and Methods. From our data, we found an average of 242 duplicated sequences among enzymes and 72 among TFs per genome, i.e., 3 times more duplicated enzymes than duplicated TFs. When the number of duplicated sequences in relation to the total enzymes and TFs per genome was expressed as a proportion, we found that on average 28% of total enzymes arise by a duplication process, whereas in the case of TFs almost 40% emerge by duplications. To determine whether there was a correlation between the duplicates and genome size, Spearman’s rank correlation test was applied. The occurrence of duplicate enzymes strongly correlated with genome size, with a Spearman coefficient of 0.94 (*p*-value <2.2e^−16^), while the Spearman’s rank correlation for duplicate TFs was 0.84 (*p*-value <2.2e^−16^); in both cases, there was a strong correlation between the variation in the number of duplicate sequences and genome size. The abundance of the duplicated sequences of enzymes and TFs grows as the genome size increases; where the best function fitted was a power-law with *R* ^2^ values of 0.9 for enzymes and 0.8 for TFs, and the exponent in the power-law fitting function of duplicated enzymes was 1.5, while that for TFs was 2.28 (Figure 5).

By analyzing the rate of increments of redundant enzymes and TFs, which were derived from the power-law function fitted to the data, it was found that the rates of duplicates were positive for both enzymes and TFs. As the size of the genome increased in organisms, a greater increment of duplicated sequences for TFs than for enzymes was observed. Thus, the TFs and their regulatory interactions have a greater plasticity and robustness than the enzymes and their metabolic interactions, which have a limited redundancy, suggesting a limited robustness and adaptability to external factors [29]. Higher values in the increment of duplicated sequences in TFs raise the possibility of generation of functional divergence, which can then lead to integration of new molecules into existing circuits in transcriptional regulation networks or can create new ones [28]. Finally, it has been reported that massive duplication followed by shuffling and probably HGT, all have significant influence on the evolution on the architecture of regulatory networks in bacteria [30]. Therefore, only 64 (13.85%) out of 462 clusters of paralogous detected in the total proteome of *E. coli* K12, and 106 (21.85%.) out of 485 clusters in *Bacillus subtilis*, contain at least one member identified as consequence of HGT [30], suggesting that duplications impact more significantly the diversity of metabolic and regulatory networks in prokaryotes than HGT events, as previously described.

## Discussion

To understand the metabolic and regulatory diversity in prokaryotes, 794 non-redundant genomes were exhaustively analyzed. Enzymes and TFs were identified based on databases annotations (KEGG, COGs, PFAM and Superfamily) and HMM searches. The analysis showed that the increment rate in the number of enzymes is negative, i.e., it diminishes with increasing genome size, and the number of acquired enzymes is always lower in large genomes than in small ones. The loss of enzymatic diversity is offset by an increase in the ability of regulation to allow a better acquisition of matter and processing of energy. On the contrary, a positive incremental changes rate of TFs was identified. The increases in the number of TFs coordinate and couple the expression of most genes and cellular functions, increasing as the metabolic and regulatory interaction networks in prokaryotes become more complex. The inverse behavior of the incremental change rate of enzymes and TFs leads to an intersection of the derivative at 9,659 genes, suggesting that this is the genome size to which a prokaryotic organism can grow while maintaining maximum metabolic variability at a minimal regulation cost, after this point the regulatory complexity increases faster than the metabolic complexity. TFs have a low number of duplications, in contrast to the apparent high number of duplications associated with enzymes. Despite the greater number of duplicated enzymes versus TFs, the rate at which duplicates appear is higher in TFs than enzymes. One of the probable strategies of bacterial and archaeal organisms to contend against changing environments is increasing the number of multifunctional enzymes, which can confer to the cell capacities of adaptation to different ecological niches. The ability of multifunctional enzymes to be less sensitive to changes in the variables of the ecological niche and their decreased regulation, which allows them to “rewire” their genetic circuits, is more apparent in *Bacteria*, which are found in environments with greater fluctuations than in *Archaea*. Overall the analyses presented here will not only contribute to improve our understanding of the design on metabolism and regulation of gene expression but also to support the basis for a comprehensive understanding of how prokaryotes are evolving and modeling their metabolic and regulatory networks. Although we understand that enzymes and TFs analyzed in this work were detected by computational methods and database annotations and probably we do not cover all the possible universe of these proteins (included those experimentally characterized), observations discussed in this study should be valid for a wide-range of prokaryotes as for most of other genomic studies [13], [14], [31].

## Material and Methods

### Proteomes Analyzed

The complete list of bacterial and archaeal genomes evaluated was downloaded from the NCBI ftp server (ftp://ncbi.nlm.nih.gov/genomes). We considered annotated genes as those genes with ORFs that encode predicted protein sequences (the proteome) in all organisms. In order to exclude any bias associated with overrepresentation of organisms that have been completely sequenced and are associated with particular divisions, non-redundant genomes were considered. In this work, we refer to non-redundant genomes as representative bacterial and archaeal species (Table S2). In brief, through the concatenation of 21 conserved proteins across diverse sequenced genomes [32], a single data set for phylogenetic analysis was constructed [33]. By eliminating genomes located more closely together on a phylogenetic tree, 794 genomes phylogenetically distant from each other were obtained, including 705 corresponding to *Bacteria* and 89 to *Archaea*. The phylogenetic tree reconstruction was based on a maximum likelihood method and was taken from the database of the Computational Genomics Group (http://www.ibt.unam.mx/biocomputo).

### Identification of Enzymatic Proteins

For each protein sequence, we identified the annotation of an E.C. class number, for which all the enzymes have been functionally classified, by using the KEGG database [34]. Then, for each protein associated with an E.C. class, the presence of both functional and structural domains, based on PFAM [35], COG [36], and Superfamily [18] assignments, were defined. Therefore, only proteins that exhibited domains in the three databases were considered in this study. Although our inclusion criterion was quite strict, we were only interested in enzymatic sequences with identified metabolic contexts and functional assignments (Table S3 and S4).

### Identification of TFs

To identify the repertoire of TFs in all bacterial and archaeal sequenced genomes, we used a combination of information sources and bioinformatics tools. We identified and evaluated all the TFs in three bacterial models, *E. coli* K-12, *Bacillus subtilis*, and *Corynebacterium glutamicum*, from three different databases, RegulonDB version 6.0 [37], DBTBS version 5.0 [38], and Coryregnet version 4.0 [39], and their domain assignments were obtained from the Superfamily database. Posteriorly, associated TFs were identified in all the complete genomes, based on specific hidden Markov model searches and from the regulators deposited in the DBD database and Superfamily database. Those with an E-value of less than 10e^−03^ and a coverage ≥60% relative to the model were considered in our analysis (Table S3 and S4).

### Evaluation of Duplication Events

Paralogous protein identification was carried out under the criteria previously described by Pushker et al., [40], [41], [42], by which paralogues are defined as protein-coding sequences within a fully sequenced genome with ≥30% sequence identity, ≥60% coverage, and an E-value cutoff of 10e^−5^. Therefore, for each single proteome, a BlastP [43] all-against-all search was performed, selecting sequences that satisfied the criteria described above. Once duplicated sequences in each genome were identified, we cross-checked the information with the list of enzymatic sequences of each organism in order to identify those enzymes that came from duplication events.

### Statistical Analysis

The correlation between genome size, measured in number of ORFs, and enzymes, TFs, and duplicated proteins was calculated using Spearman’s rank correlation coefficient. Standard residual plot analyses to determine whether a distribution was linear were also performed. Finally, a Wilcoxon test, for comparing means, and linear regression analyses were performed using the R programming language for statistical analysis [44].

## Supporting Information

Figure S1 **Sliding-window boxplot of detected enzymes in Bacteria and Archaea. 11 windows with a length of 836 ORFs were considered.** 11 windows with a length of 836 ORFs were considered. In *x* axis is the number of windows. In *y* axis is the number of enzymes. The mean of each window is displayed with a red circle and the fitted power-law function is shown with a black line. The number of windows was calculated by using the Sturges's formula, which is used to group many different values in equal classes: *k* = 1+ log2N where *k* is the number of equal classes and N the number of data, rounding to the nearest integer, the *k* value. Then, the width of classes is determined with the following equation: *c* = R/*k* where R = high value – low value (Genome size). Values resulting from the application of the above formulas were *k* = 11 and *c* = 836, thus 11 windows without overlaps were used with a width of 836 ORFs. Subsequently, the number of windows was increased in 11, obtaining 22 windows with a width of 418 ORFs. This procedure was performed three times, increasing the number of windows in 11. Based on this approach three more sets of 33, 44 and 55 windows with a width of 278, 209 and 167 ORFs, respectively, were obtained.
(TIF)

Figure S2 **top) Comparing the percentage of enzymes into eleven size classes.** As in figure S1, 11 windows with a length of 836 ORFs were considered. In *x* axis is the number of genome sizes classes. In *y* axis is the percentage of enzymes per class. Numbers upper each bar denotes the average of enzymes per class. In light gray are the bacterial genomes, dark gray are the bacterial subset and in white color are the archaea genomes. Figure S2. bottom) Average of metabolic KEGG pathways per genome. In *x* axis are the functional KEGG categories. In y axis are the mean of metabolic KEGG pathways by organism. In light gray are the bacterial genomes, dark gray are the bacterial subset and in white color are the archaea genomes.
(TIF)

Figure S3 **Percentage of enzymes associated with two or more metabolic pathways.** In *x* axis is the number of pathways. In *y* axis is the percentage of enzymes associated to each pathway class. In light gray are the bacterial genomes, dark gray are the bacterial subset and in white color are the archaeal genomes.
(TIF)

Figure S4 **Percentage of enzymes classified by their E.C. numbers.** In *x* axis are the six E.C. classes. In *y* axis is the percentage of enzymes associated to each class. In light gray are the bacterial genomes, dark gray are the bacterial subset and in white color are the archaeal genomes.
(TIF)

Figure S5 **Sliding-window boxplot of detected TFs in Bacteria and Archaea.** 11 windows with a length of 836 ORFs were considered. In *x* axis is number of windows. In *y* axis is the number of TFs. The mean of each window is displayed with a red circle and the fitted power-law function is shown with a black line.
(TIF)

Table S1 **Power-law functions fitted to different sliding-windows.** Nomenclature is as follows: Column 1 denotes the number of windows considered and their size in ORFs; Columns 2 and 3 shown power-law function and R^2^ associated to enzymes; and columns 4 and 5 shown power-law function and R^2^ associated to TFs.
(DOCX)

Table S2 **Genomes analyzed in this work.** 794 bacterial and archaeal genomes were considered in this work.
(DOCX)

Table S3 **Prokaryotic genomes, Transcription factors, Enzymes and their respective annotations are provided.** The information of genomes, transcription factors and enzymes is placed in a tabular format. In the case of enzymes the directory DatasetS2_enzymes contains 794 files, one per genome. The information is organized as in follows: KEGGID: identifier of gene from KEGG database; NCBIGI: identifier from NCBI database; ECS: E.C.s numbers from KEGG; PfamID: identifier of Pfam domain; COGID: identifier of COG domain; SFamilyID: identifier of superfamily domain. For the case of TFs the directory DatasetS2_tfs contains 794 files, one per genome. Nomenclature is as follows: KEGGID: identifier of gene from KEGG database; NCBIGI: identifier from NCBI database; PfamID: identifier of Pfam domain; SFamilyID: identifier of superfamily domain. The file orgsIdName.txt contains the names of the organism and their KEGG-ID identifiers.
(7z) Click here for additional data file.

Table S4 **Scripts in Perl to obtain the equivalents GI-NCBI or ID-KEGG identifiers from the KEGG database.**
(ZIP)
