# Supplementary figures and images for: Increments and Duplication Events of Enzymes and Transcription Factors Influence Metabolic and Regulatory Diversity in Prokaryotes
Source: PLoS One. 2013 Jul 29;8(7):e69707. doi: 10.1371/journal.pone.0069707 (PMC3726781; doi:10.1371/journal.pone.0069707)

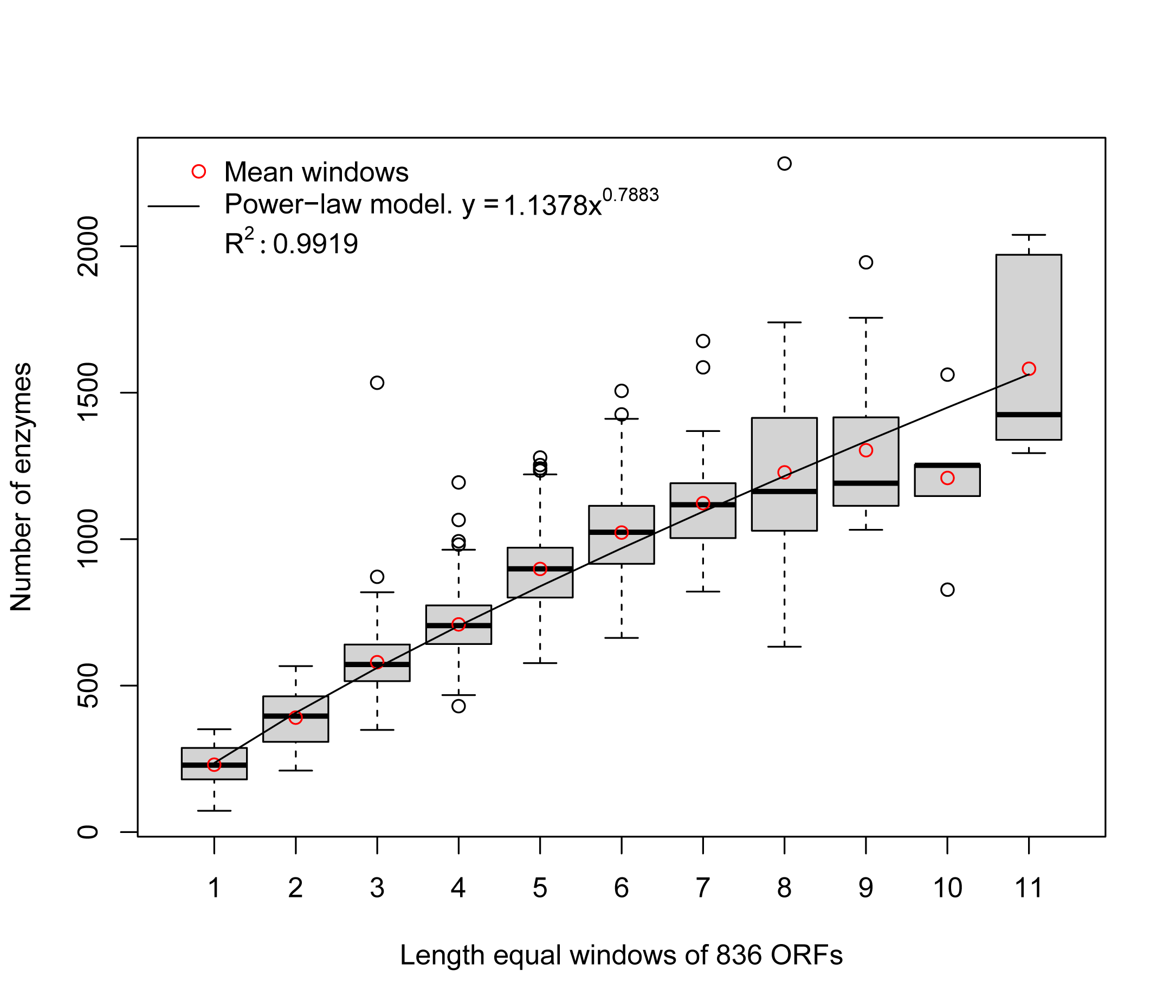

Supplement: Figure S1 — Sliding-window boxplot of detected enzymes in Bacteria and Archaea. 11 windows with a length of 836 ORFs were considered. 11 windows with a length of 836 ORFs were considered. In x axis is the number of windows. In y axis is the number of enzymes. The mean of each window is displayed with a red circle and the fitted power-law function is shown with a black line. The number of windows was calculated by using the Sturges's formula, which is used to group many different values in equal classes: k = 1+ log2N where k is the number of equal classes and N the number of data, rounding to the nearest integer, the k value. Then, the width of classes is determined with the following equation: c = R/k where R = high value – low value (Genome size). Values resulting from the application of the above formulas were k = 11 and c = 836, thus 11 windows without overlaps were used with a width of 836 ORFs. Subsequently, the number of windows was increased in 11, obtaining 22 windows with a width of 418 ORFs. This procedure was performed three times, increasing the number of windows in 11. Based on this approach three more sets of 33, 44 and 55 windows with a width of 278, 209 and 167 ORFs, respectively, were obtained. (TIF) [file pone.0069707.s001.tif]

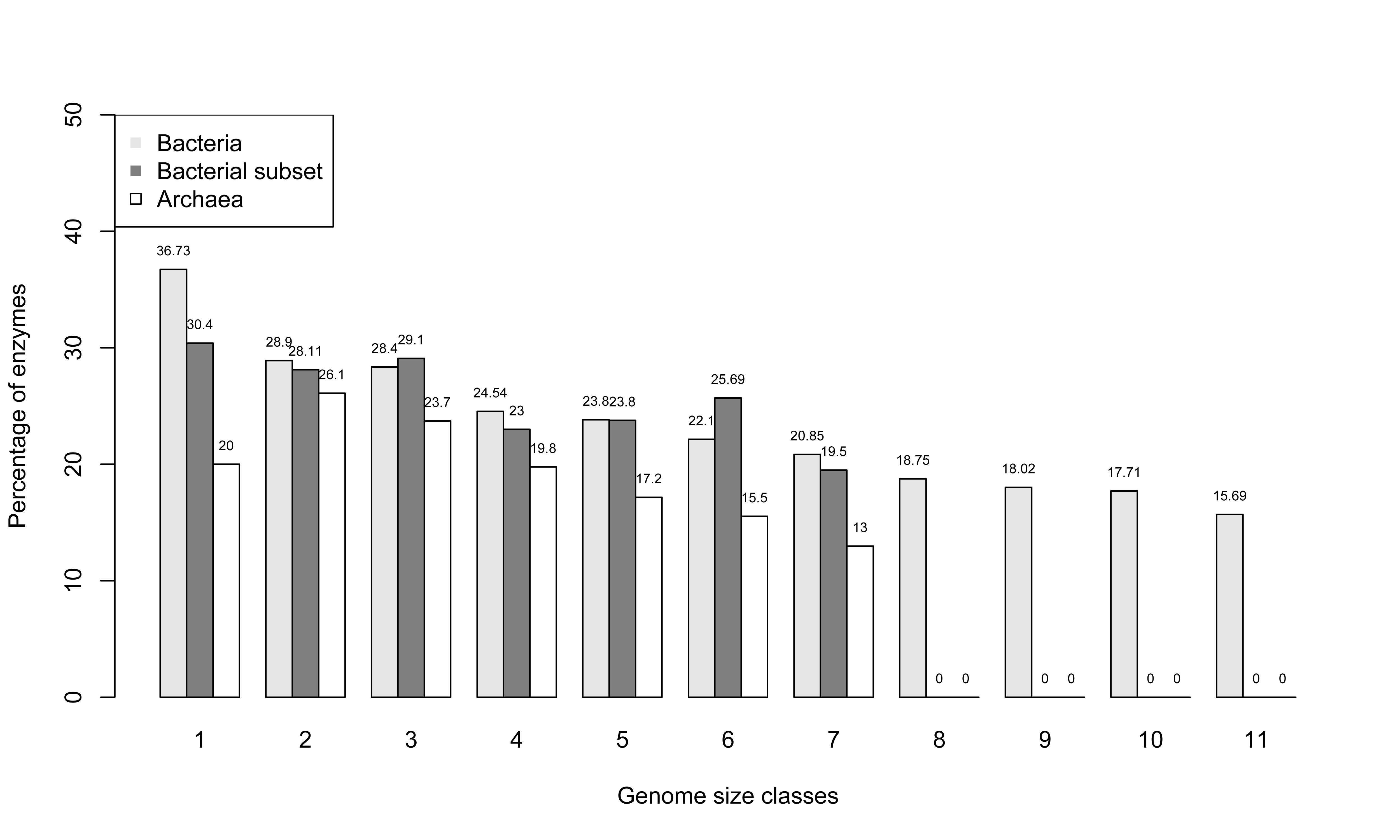

Supplement: Figure S2 — top) Comparing the percentage of enzymes into eleven size classes. As in figure S1, 11 windows with a length of 836 ORFs were considered. In x axis is the number of genome sizes classes. In y axis is the percentage of enzymes per class. Numbers upper each bar denotes the average of enzymes per class. In light gray are the bacterial genomes, dark gray are the bacterial subset and in white color are the archaea genomes. Figure S2. bottom) Average of metabolic KEGG pathways per genome. In x axis are the functional KEGG categories. In y axis are the mean of metabolic KEGG pathways by organism. In light gray are the bacterial genomes, dark gray are the bacterial subset and in white color are the archaea genomes. (TIF) [file pone.0069707.s002.tif]

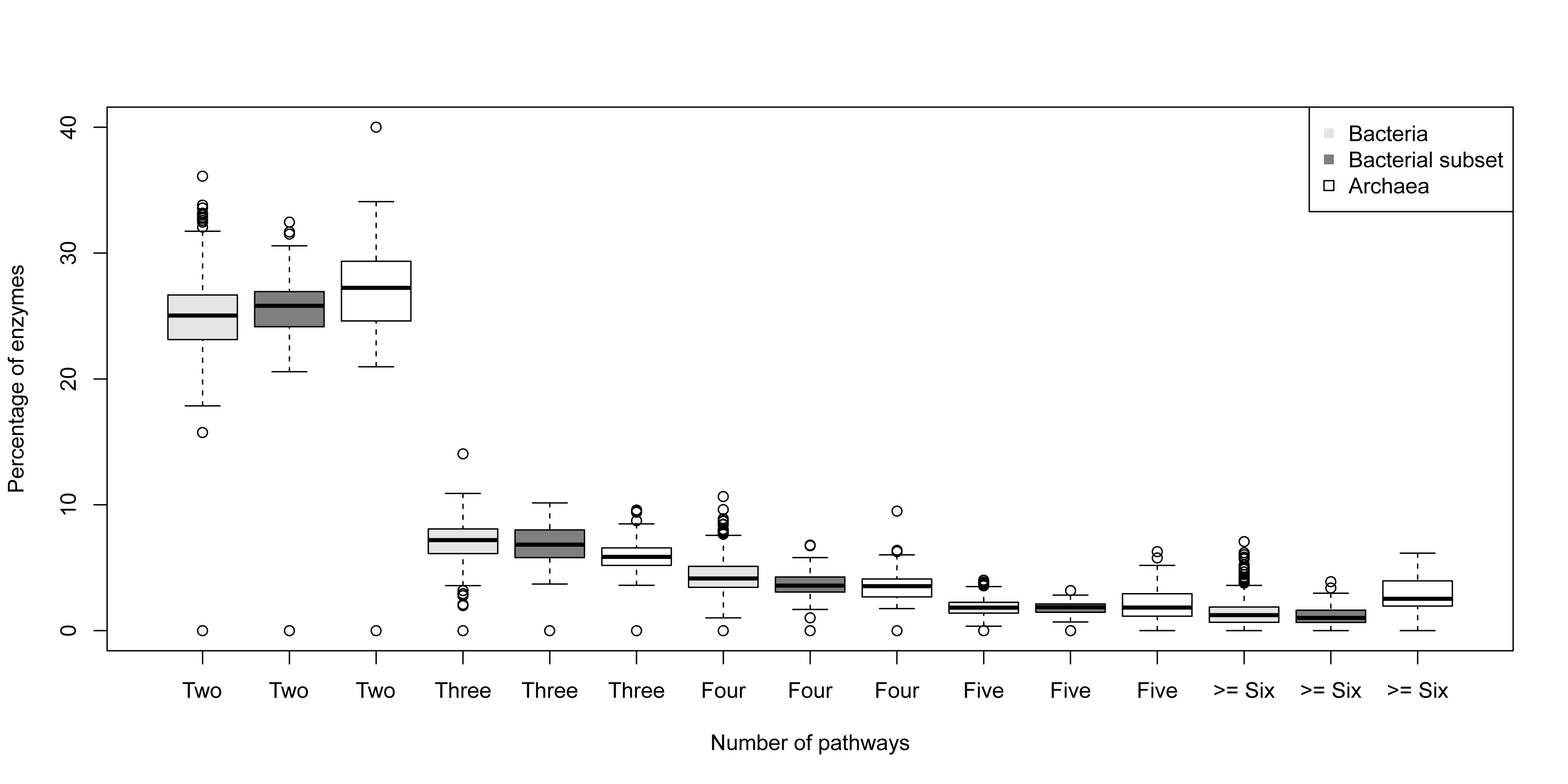

Supplement: Figure S3 — Percentage of enzymes associated with two or more metabolic pathways. In x axis is the number of pathways. In y axis is the percentage of enzymes associated to each pathway class. In light gray are the bacterial genomes, dark gray are the bacterial subset and in white color are the archaeal genomes. (TIF) [file pone.0069707.s003.tif]

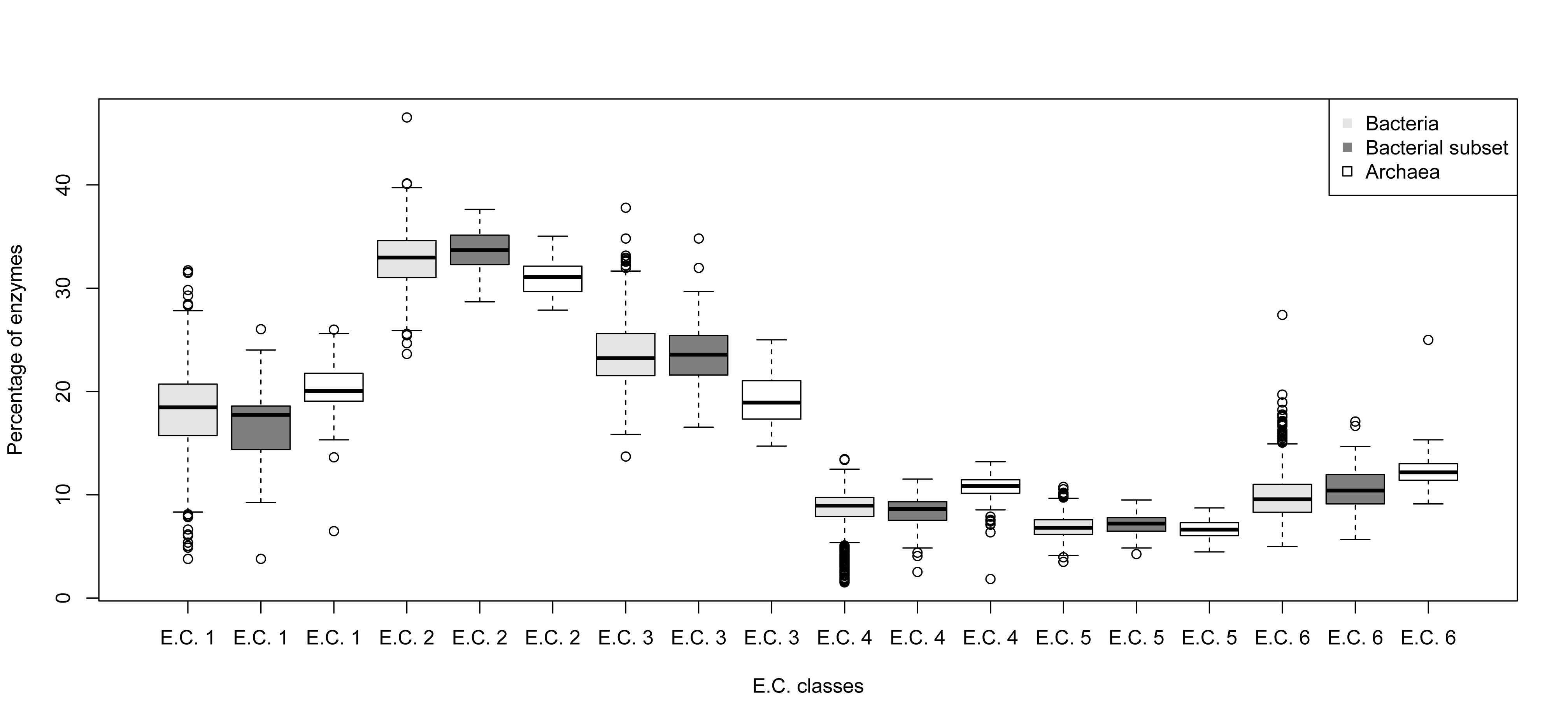

Supplement: Figure S4 — Percentage of enzymes classified by their E.C. numbers. In x axis are the six E.C. classes. In y axis is the percentage of enzymes associated to each class. In light gray are the bacterial genomes, dark gray are the bacterial subset and in white color are the archaeal genomes. (TIF) [file pone.0069707.s004.tif]

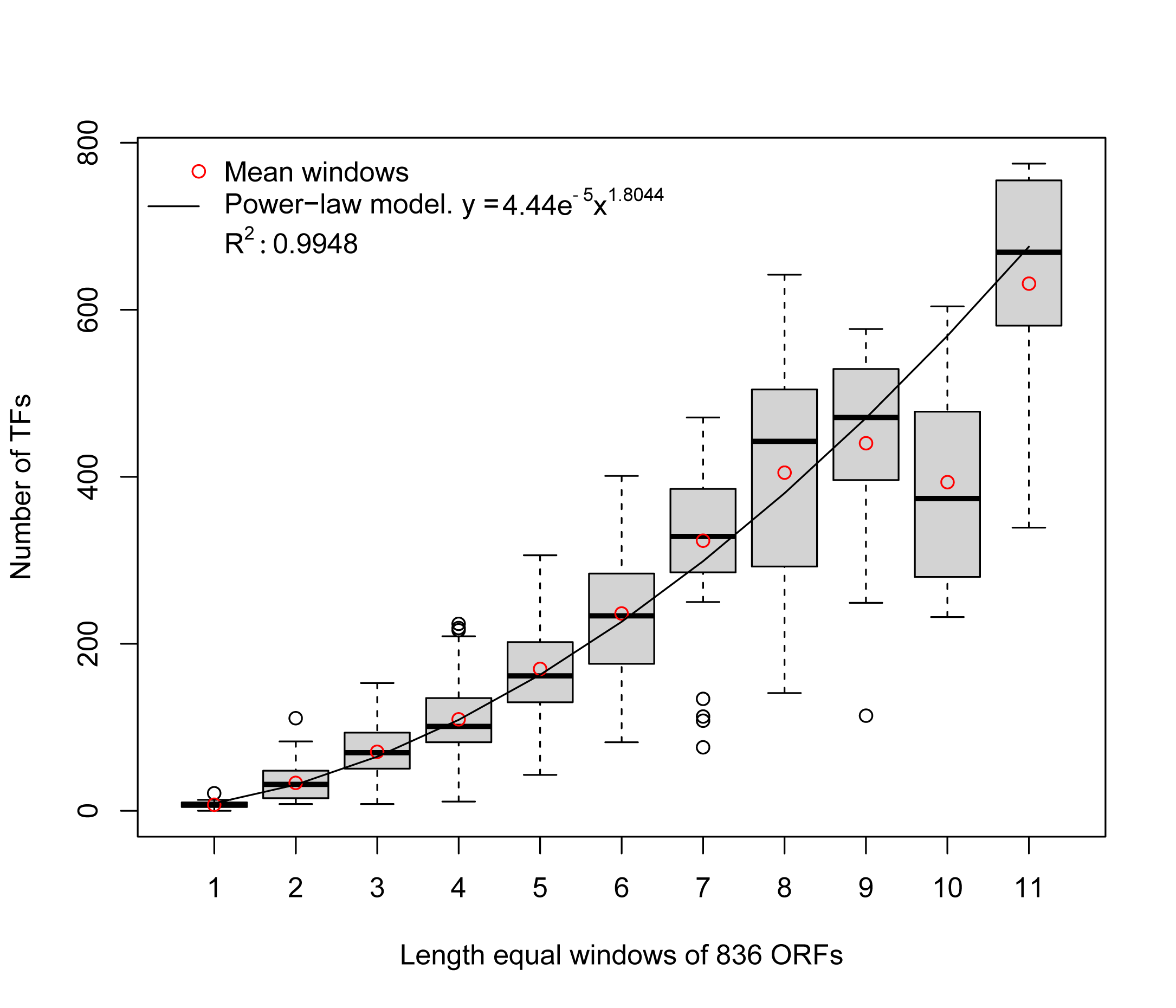

Supplement: Figure S5 — Sliding-window boxplot of detected TFs in Bacteria and Archaea. 11 windows with a length of 836 ORFs were considered. In x axis is number of windows. In y axis is the number of TFs. The mean of each window is displayed with a red circle and the fitted power-law function is shown with a black line. (TIF) [file pone.0069707.s005.tif]
