# Supplementary material for: Increments and Duplication Events of Enzymes and Transcription Factors Influence Metabolic and Regulatory Diversity in Prokaryotes
Source: PLoS One. 2013 Jul 29;8(7):e69707. doi: 10.1371/journal.pone.0069707 (PMC3726781; doi:10.1371/journal.pone.0069707)
Supplement: Table S1 — Power-law functions fitted to different sliding-windows. Nomenclature is as follows: Column 1 denotes the number of windows considered and their size in ORFs; Columns 2 and 3 shown power-law function and R2 associated to enzymes; and columns 4 and 5 shown power-law function and R2 associated to TFs. (DOCX) [file pone.0069707.s006.docx]

**Table S1. Power-law functions fitted to different sliding-windows.**

| **Window (Length size)** | **Enzymes** | | **TFs** | |
| --- | --- | --- | --- | --- |
|  | **Power-law functions** | **R^2^** | **Power-law functions** | **R^2^** |
| 11 windows (836 ORFs). | y = 1.1378x^0.7883. | 0.9919 | y = 4.4400e-5x^1.8044 | 0.9948 |
| 22 windows (418 ORFs) | y = 1.1954x^0.7868. | 0.9380 | y = 0.000015759x^1.935 | 0.9806 |
| 33 windows (278 ORFs) | y = 1.2880x^0.7781. | 0.9531 | y = 0.000155319x^1.6655 | 0.9647 |
| 44 windows (209 ORFs) | y = 1.8073x^0.7396. | 0.9585 | y = 9.7974e-05x^1.7226 | 0.9677 |
| 55 windows (167 ORFs) | y = 1.2517x^0.7840. | 0.9493 | y = 0.00013218x^1.6897 | 0.9691 |

Nomenclature is as follow: Column 1 denotes the number of windows considered and their size in ORFs; Columns 2 and 3 shown power-law function and R^2^ associated to enzymes; and columns 4 and 5 shown power-law function and R^2^ associated to TFs.
