# Supplementary material for: Increments and Duplication Events of Enzymes and Transcription Factors Influence Metabolic and Regulatory Diversity in Prokaryotes
Source: PLoS One. 2013 Jul 29;8(7):e69707. doi: 10.1371/journal.pone.0069707 (PMC3726781; doi:10.1371/journal.pone.0069707)
Supplement: Table S2 — Genomes analyzed in this work. 794 bacterial and archaeal genomes were considered in this work. (DOCX) [file pone.0069707.s007.docx]

**Organisms considered in this analysis.**

**Archaea**: *Nanoarchaeum equitans; Methanothermus fervidus; Thermosphaera aggregans; Ignicoccus hospitalis; Methanocaldococcus infernus; Desulfurococcus kamchatkensis; Thermoplasma acidophilum; Methanococcus aeolicus; Acidilobus saccharovorans; Thermoplasma volcanium; Methanosphaera stadtmanae; Picrophilus torridus; Aciduliprofundum boonei; Staphylothermus marinus; Methanocaldococcus fervens; Staphylothermus hellenicus; Candidatus Korarchaeum cryptofilum; Hyperthermus butylicus; Methanococcus vannielii; Methanopyrus kandleri; Methanosaeta thermophila; Aeropyrum pernix; Methanococcus voltae; Methanococcus maripaludis S2; Methanocorpusculum labreanum; Methanocaldococcus vulcanius; Methanothermobacter marburgensis; Methanocaldococcus jannaschii; Pyrococcus abyssi; Methanococcus maripaludis C7; Methanobrevibacter smithii ATCC 35061; Nitrosopumilus maritimus; Methanocaldococcus sp. FS406-22; Methanococcus maripaludis C5; Archaeoglobus profundus; Methanococcus maripaludis C6; Methanothermobacter thermautotrophicus; Thermofilum pendens; Ignisphaera aggregans; Pyrococcus horikoshii; Caldivirga maquilingensis; Thermoproteus neutrophilus; Thermococcus onnurineus; Pyrobaculum islandicum; Methanohalophilus mahii; Thermococcus sibiricus; Pyrococcus furiosus; Pyrobaculum calidifontis; Thermococcus gammatolerans; Methanobrevibacter ruminantium; Sulfolobus acidocaldarius; Methanohalobium evestigatum; Metallosphaera sedula; Methanococcoides burtonii; Pyrobaculum arsenaticum; Thermococcus kodakaraensis; Archaeoglobus fulgidus; Methanoregula boonei; Ferroglobus placidus; Methanoculleus marisnigri; Vulcanisaeta distributa; Pyrobaculum aerophilum; Sulfolobus islandicus M.14.25; Halobacterium sp. NRC-1; Haloquadratum walsbyi; Candidatus Methanosphaerula palustris; Halobacterium salinarum R1; Methanoplanus petrolearius; Natronomonas pharaonis; Sulfolobus tokodaii; Sulfolobus islandicus Y.N.15.51; Sulfolobus islandicus Y.G.57.14; Sulfolobus islandicus L.D.8.5; Sulfolobus solfataricus; Halorhabdus utahensis; Methanocella paludicola; Uncultured methanogenic archaeon RC-I; Methanospirillum hungatei; Halomicrobium mukohataei; Methanosarcina mazei; Halorubrum lacusprofundi; Methanosarcina barkeri; Halalkalicoccus jeotgali; Halogeometricum borinquense; Haloferax volcanii; Natrialba magadii; Haloarcula marismortui; Methanosarcina acetivorans; Haloterrigena turkmenica*.

**Bacteria**: *Candidatus Carsonella ruddii; Buchnera aphidicola Cc; Mycoplasma genitalium; Candidatus Phytoplasma mali; Buchnera aphidicola Bp; Mycoplasma hominis; Buchnera aphidicola Sg; Candidatus Riesia pediculicola; Buchnera aphidicola APS; Blattabacterium sp. (Periplaneta americana); Candidatus Blochmannia floridanus; Blattabacterium sp. (Blattella germanica); Baumannia cicadellinicola; Candidatus Blochmannia pennsylvanicus; Ureaplasma parvum serovar 3 ATCC 700970; Wigglesworthia glossinidia; Mycoplasma arthritidis; Mycoplasma mobile; Ureaplasma urealyticum serovar 10 ATCC 33699; Mycoplasma hyorhinis; Mycoplasma synoviae; Mesoplasma florum; Candidatus Phytoplasma australiense; Mycoplasma pneumoniae; Mycoplasma crocodyli; Mycoplasma hyopneumoniae 232; Mycoplasma conjunctivae; Mycoplasma gallisepticum; Mycoplasma bovis PG45; Mycoplasma pulmonis; Mycoplasma fermentans JER; Wolbachia wBm; Tropheryma whipplei Twist; Mycoplasma agalactiae 5632; Borrelia turicatae; Borrelia hermsii; Rickettsia prowazekii; Rickettsia typhi; Candidatus Azobacteroides pseudotrichonymphae; Mycoplasma leachii; Neorickettsia risticii; Chlamydia muridarum; Chlamydia trachomatis A/HAR-13; Anaplasma centrale; Ehrlichia canis; Borrelia garinii; Chlamydophila abortus; Neorickettsia sennetsu; Candidatus Vesicomyosocius okutanii; Anaplasma marginale St. Maries; Ehrlichia ruminantium Welgevonden (France); Candidatus Ruthia magnifica; Rickettsia massiliae; Chlamydophila caviae; Chlamydophila felis; Mycoplasma mycoides subsp. mycoides SC PG1; Treponema pallidum subsp. pallidum Nichols; Mycoplasma penetrans; Rickettsia canadensis; Ehrlichia chaffeensis; Candidatus Liberibacter asiaticus; Chlamydophila pneumoniae AR39; Bartonella quintana; Orientia tsutsugamushi Boryong; Candidatus Liberibacter solanacearum; Wolbachia wMel; Cyanobacterium UCYN-A; Borrelia afzelii; Rickettsia akari; Gardnerella vaginalis; Anaplasma phagocytophilum; Wolbachia pipientis; Dichelobacter nodosus; Bartonella bacilliformis; Borrelia duttonii; Candidatus Amoebophilus asiaticus; Lawsonia intracellularis; Atopobium parvulum; Candidatus Pelagibacter ubique; Cryptobacterium curtum; Gardnerella vaginalis ATCC 14019; Acholeplasma laidlawii; Rickettsia rickettsii Iowa; Helicobacter mustelae; Streptobacillus moniliformis; Dehalococcoides sp. CBDB1; Rickettsia bellii OSU 85-389; Coprothermobacter proteolyticus; Bartonella henselae; Polynucleobacter necessarius; Rickettsia felis; Lactobacillus delbrueckii ATCC 11842; Elusimicrobium minutum; Campylobacter lari; Aquifex aeolicus; Bifidobacterium animalis subsp. lactis Bl-04; Clostridiales genomosp. BVAB3; Dehalococcoides ethenogenes; Thermocrinis albus; Mycobacterium leprae Br4923; Lactobacillus helveticus; Helicobacter acinonychis; Hydrogenobaculum sp. Y04AAS1; Bifidobacterium adolescentis; Borrelia burgdorferi B31; Dehalogenimonas lykanthroporepellens; Helicobacter felis; Campylobacter hominis ATCC BAA-381; Oenococcus oeni; Helicobacter pylori B8; Streptococcus thermophilus LMD-9; Prochlorococcus marinus MED4; Haemophilus ducreyi; Campylobacter fetus; Sulfurihydrogenibium sp. YO3AOP1; Sulfurihydrogenibium azorense; Nautilia profundicola; Arcanobacterium haemolyticum; Campylobacter jejuni subsp. doylei 269.97; Thermanaerovibrio acidaminovorans; Olsenella uli; Dictyoglomus turgidum; Fervidobacterium nodosum; Chlorobium phaeovibrioides; Francisella tularensis subsp. holarctica LVS; Pediococcus pentosaceus; Streptococcus uberis; Bartonella grahamii; Bifidobacterium bifidum S17; Haemophilus influenzae 86-028NP (nontypeable); Anaerococcus prevotii; Prochlorococcus marinus MIT9312; Finegoldia magna; Leuconostoc citreum; Lactobacillus johnsonii NCC 533; Nitratiruptor sp. SB155-2; Lactobacillus fermentum; Veillonella parvula; Prochlorococcus marinus MIT 9211; Thermotoga maritima; Lactobacillus acidophilus NCFM; Helicobacter hepaticus; Aminobacterium colombiense; Lactobacillus sakei; Thermosipho melanesiensis; Prochlorococcus marinus SS120; Zymomonas mobilis subsp. mobilis NCIMB 11163; Moraxella catarrhalis; Hydrogenobacter thermophilus; Petrotoga mobilis; Lactobacillus reuteri DSM 20016; Rothia mucilaginosa; Prochlorococcus marinus MIT 9515; Mobiluncus curtisii; Dictyoglomus thermophilum; Leuconostoc gasicomitatum; Francisella philomiragia; Prochlorococcus marinus AS9601; Campylobacter curvus; Thermotoga neapolitana; Thermosipho africanus; Waddlia chondrophila; Streptococcus mutans UA159; Acidimicrobium ferrooxidans DSM 10331; Riemerella anatipestifer; Neisseria lactamica; Haemophilus somnus 2336; Prochlorococcus marinus MIT 9215; Campylobacter concisus 13826; Streptococcus equi subsp. equi 4047; Chlorobium chlorochromatii; Leuconostoc mesenteroides; Lactobacillus salivarius; Pasteurella multocida; Corynebacterium kroppenstedtii; Haemophilus parasuis; Corynebacterium urealyticum; Lactobacillus crispatus; Acidaminococcus fermentans; Leifsonia xyli xyli CTCB07; Candidatus Protochlamydia amoebophila; Thermodesulfovibrio yellowstonii; Thermotoga lettingae; Wolinella succinogenes; Chlorobaculum parvum NCIB 8327; Coxiella burnetii Dugway 5J108-111; Persephonella marina; Streptococcus gordonii; Macrococcus caseolyticus; Fusobacterium nucleatum; Polynucleobacter sp. QLW-P1DMWA-1; Actinobacillus succinogenes; Ammonifex degensii; Pelodictyon luteolum; Bartonella tribocorum; Porphyromonas gingivalis ATCC 33277; Streptococcus dysgalactiae; Sulfurimonas denitrificans; Calditerrivibrio nitroreducens; Corynebacterium pseudotuberculosis; Kosmotoga olearia; Corynebacterium jeikeium; Lactobacillus amylovorus; Streptococcus agalactiae 2603 (serotype V); Leuconostoc kimchii; Bifidobacterium dentium; Akkermansia muciniphila; Actinobacillus pleuropneumoniae AP76 (serotype 7); Candidatus Hamiltonella defensa; Acidothermus cellulolyticus; Candidatus Desulforudis audaxviator; Capnocytophaga ochracea; Streptococcus suis 05ZYH33; Prochlorococcus marinus NATL1A; Thiomicrospira crunogena; Thermosediminibacter oceani; Rothia dentocariosa; Lactobacillus brevis; Aggregatibacter aphrophilus; Aggregatibacter actinomycetemcomitans D11S-1; Leptotrichia buccalis; Streptococcus gallolyticus UCN34; Thermus thermophilus HB8; Micrococcus luteus; Chlorobaculum tepidum; Arcobacter butzleri; Sulfurospirillum deleyianum; Streptococcus sanguinis; Thermoanaerobacter italicus; Corynebacterium diphtheriae; Acetohalobium arabaticum; Prevotella melaninogenica; Brachyspira pilosicoli; Synechococcus sp. CC9902; Syntrophothermus lipocalidus; Clostridium novyi; Prosthecochloris aestuarii; Methylotenera mobilis; Halothermothrix orenii; Propionibacterium acnes SK137; Streptococcus pneumoniae 670-6B; Desulfovibrio desulfuricans ATCC 27774; Kocuria rhizophila; Halothiobacillus neapolitanus; Mannheimia succiniciproducens; Oceanithermus profundus; Deferribacter desulfuricans SSM1; Propionibacterium freudenreichii; Psychrobacter sp. PRwf-1; Halorhodospira halophila; Flavobacterium psychrophilum; Bifidobacterium longum subsp. infantis ATCC 15697; Granulibacter bethesdensis; Chlorobium limicola; Clostridium tetani E88; Sulfurovum sp. NBC37-1; Staphylococcus carnosus; Nitrosomonas europaea; Moorella thermoacetica; Chlorobium phaeobacteroides BS1; Methylacidiphilum infernorum; Thermosynechococcus elongatus; Staphylococcus lugdunensis; Syntrophomonas wolfei; Lactococcus lactis subsp. cremoris SK11; Psychrobacter cryohalolentis; Jonesia denitrificans; Staphylococcus saprophyticus; Sodalis glossinidius; Synechococcus sp. WH8102; Staphylococcus epidermidis RP62A; Desulfohalobium retbaense; Synechococcus sp. WH7803; Flavobacteriaceae bacterium; Synechococcus sp. RCC307; Candidatus Puniceispirillum marinum; Corynebacterium aurimucosum; Nitrosomonas eutropha; Kytococcus sedentarius; Clostridium sticklandii; Thermoanaerobacter tengcongensis; Thermoanaerobacterium thermosaccharolyticum; Desulfurivibrio alkaliphilus; Carboxydothermus hydrogenoformans; Idiomarina loihiensis; Kangiella koreensis; Brachyspira hyodysenteriae; Synechococcus sp. CC9605; Chlorobium phaeobacteroides DSM 266; Synechococcus elongatus PCC7942; Gluconobacter oxydans; Caldicellulosiruptor bescii; Neisseria gonorrhoeae NCCP11945; Caldicellulosiruptor saccharolyticus; Parvularcula bermudensis; Staphylococcus haemolyticus; Croceibacter atlanticus; Pelodictyon phaeoclathratiforme; Chloroherpeton thalassium; Methylobacillus flagellatus; Alcanivorax borkumensis; Synechococcus sp. JA-3-3Ab; Prevotella ruminicola; Methylotenera sp. 301; Eubacterium eligens; Slackia heliotrinireducens; Treponema denticola; Sulfuricurvum kujiense; Nitrosospira multiformis; Brachyspira murdochii; Thiobacillus denitrificans; Anoxybacillus flavithermus; Thermobaculum terrenum; Xylella fastidiosa 9a5c; Alkaliphilus oremlandii; Thermomicrobium roseum; Thioalkalivibrio sp. K90mix; Synechococcus sp. JA-2-3B'a(2-13); Rhodothermus marinus DSM 4252; Alkalilimnicola ehrlichei; Clostridium perfringens ATCC 13124; Ilyobacter polytropus; Staphylococcus aureus NCTC8325; Synechococcus sp. CC9311; Gallionella capsiferriformans; Natranaerobius thermophilus; Nitrosococcus watsonii; Methylovorus glucosetrophus SIP3-4; Pelotomaculum thermopropionicum; Truepera radiovictrix; Leptospira borgpetersenii L550; Thermincola potens JR; Methylococcus capsulatus; Lactobacillus rhamnosus Lc 705; Denitrovibrio acetiphilus; Sideroxydans lithotrophicus; Corynebacterium efficiens; Prochlorococcus marinus MIT 9303; Heliobacterium modesticaldum; Segniliparus rotundus; Erythrobacter litoralis; Meiothermus ruber; Lactobacillus casei BL23; Exiguobacterium sibiricum; Nitrosococcus oceani; Exiguobacterium sp. AT1b; Acetobacter pasteurianus; Deinococcus geothermalis; Lactobacillus plantarum WCFS1; Maricaulis maris; Brachybacterium faecium; Eggerthella lenta; Corynebacterium glutamicum R; Alicyclobacillus acidocaldarius; Fibrobacter succinogenes; Thermobifida fusca; Listeria monocytogenes 08-5578; Xanthomonas albilineans; Clavibacter michiganensis subsp. sepedonicus; Coraliomargarita akajimensis; Nitrobacter winogradskyi; Arcobacter nitrofigilis; Tolumonas auensis; Rubrobacter xylanophilus; Acidithiobacillus ferrooxidans ATCC 23270; Kyrpidia tusciae; Syntrophus aciditrophicus; Legionella pneumophila Paris; Deinococcus radiodurans; Thiomonas intermedia; Desulfovibrio vulgaris Miyazaki F; Salinibacter ruber; Hirschia baltica; Synechococcus sp. PCC7002; Clostridium thermocellum; Sphingopyxis alaskensis; Robiginitalea biformata; Ketogulonicigenium vulgare; Allochromatium vinosum; Desulfotalea psychrophila; Laribacter hongkongensis; Bacillus selenitireducens; Enterococcus faecalis; Candidatus Desulfococcus oleovorans; Brucella suis 1330; Desulfotomaculum reducens; Desulfarculus baarsii; Thioalkalivibrio sp. HL-EbGR7; Herminiimonas arsenicoxydans; Chromohalobacter salexigens; Acinetobacter sp. ADP1; Brevundimonas subvibrioides; Symbiobacterium thermophilum; Pelobacter carbinolicus; Acidobacterium capsulatum; Bordetella avium; Clostridium cellulolyticum; Maribacter sp. HTCC2170; Geobacter sulfurreducens; Desulfomicrobium baculatum; Arthrobacter arilaitensis; Xylanimonas cellulosilytica; Geobacillus thermodenitrificans; Geobacillus sp. Y412MC61; Deinococcus deserti; Leadbetterella byssophila; Legionella longbeachae; Halomonas elongata; Clostridium botulinum B Eklund 17B; Pseudoalteromonas haloplanktis; Sphaerobacter thermophilus; Oceanobacillus iheyensis; Hyphomonas neptunium; Meiothermus silvanus; Renibacterium salmoninarum; Hyphomicrobium denitrificans; Geobacter metallireducens; Desulfovibrio vulgaris Hildenborough; Psychromonas ingrahamii; Thermobispora bispora; Acidiphilium cryptum JF-5; Rhodomicrobium vannielii; Synechocystis sp. PCC6803; Gramella forsetii; Bdellovibrio bacteriovorus; Eubacterium rectale; Parvibaculum lavamentivorans; Rhodobacter capsulatus; Shewanella amazonensis; Anabaena azollae 0708; Proteus mirabilis; Leptospira interrogans serovar Copenhageni; Geobacillus sp. Y4.1MC1; Cellulomonas flavigena; Bacillus pumilus; Geobacter lovleyi; Erwinia pyrifoliae; Sanguibacter keddieii; Pseudoalteromonas sp. SM9913; Magnetococcus sp. MC-1; Oligotropha carboxidovorans OM5 (Mississippi); Leptospira biflexa serovar Patoc Patoc 1 (Paris); Chloroflexus aggregans; Clostridium difficile 630; Cellvibrio japonicus; Shewanella denitrificans; Marivirga tractuosa; Desulfovibrio desulfuricans G20; Ferrimonas balearica; Beijerinckia indica; Edwardsiella ictaluri; Cytophaga hutchinsonii; Geobacter sp. FRC-32; Pelobacter propionicus; Desulfovibrio salexigens; Butyrivibrio proteoclasticus; Nitrosococcus halophilus; Methylocella silvestris; Saccharomonospora viridis; Vibrio cholerae O1 biovar El Tor N16961; Rhodospirillum rubrum; Bacillus cytotoxicus NVH 391-98; Clostridium acetobutylicum ATCC 824; Parabacteroides distasonis; Gluconacetobacter diazotrophicus PAl 5 (Brazil); Phenylobacterium zucineum; Shewanella loihica; Burkholderia rhizoxinica; Acinetobacter sp. DR1; Caulobacter crescentus NA1000; Bacillus amyloliquefaciens DSM 7; Clostridium phytofermentans; Aliivibrio salmonicida LFI1238; Clostridium kluyveri DSM 555; Gemmatimonas aurantiaca; Novosphingobium aromaticivorans; Dickeya dadantii Ech703; Thauera sp. MZ1T; Azoarcus sp. BH72; Clostridium botulinum Ba4; Rhodospirillum centenum; Saccharophagus degradans; Shewanella frigidimarina; Vibrio fischeri MJ11; Syntrophobacter fumaroxidans; Bacillus halodurans; Bacteroides vulgatus; Desulfotomaculum acetoxidans; Geobacter sp. M21; Bacillus clausii; Pseudomonas stutzeri; Roseobacter denitrificans; Caulobacter segnis; Sebaldella termitidis; Clostridium saccharolyticum; Acidovorax sp. JS42; Chloroflexus sp. Y-400-fl; Dechloromonas aromatica; Bacillus licheniformis ATCC 14580; Bacillus subtilis; Clostridium ljungdahlii; Bacillus atrophaeus; Mycobacterium tuberculosis CDC1551; Dinoroseobacter shibae; Beutenbergia cavernae; Spirochaeta smaragdinae; Enterobacter sp. 638; Pantoea ananatis; Tsukamurella paurometabola; Pedobacter heparinus; Silicibacter pomeroyi; Clostridium cellulovorans; Teredinibacter turnerae; Planctomyces limnophilus; Xenorhabdus bovienii; Marinobacter aquaeolei; Shewanella halifaxensis; Pseudoalteromonas atlantica; Jannaschia sp. CCS1; Yersinia pseudotuberculosis IP31758 (serotype O:1b); Nitrobacter hamburgensis; Roseiflexus castenholzii DSM13941; Rhodobacter sphaeroides ATCC 17025; Bacillus pseudofirmus; Shewanella violacea; Geobacter uraniumreducens; Leptothrix cholodnii; Stenotrophomonas maltophilia K279a; Sphingobium japonicum; Rhodopseudomonas palustris BisB5; Enterobacter cloacae SCF1; Chromobacterium violaceum; Rhodoferax ferrireducens; Gloeobacter violaceus; Starkeya novella; Vibrio splendidus; Aeromonas salmonicida; Pectobacterium wasabiae; Marinomonas sp. MWYL1; Cyanothece sp. PCC 8802; Methylibium petroleiphilum; Trichodesmium erythraeum; Cronobacter turicensis; Xanthomonas campestris pv. campestris B100; Anaeromyxobacter sp. Fw109-5; Shewanella oneidensis; Anaeromyxobacter dehalogenans 2CP-1; Xenorhabdus nematophila; Shewanella sediminis; Frankia sp. CcI3; Eubacterium limosum; Rhodococcus equi; Roseiflexus sp. RS-1; Arthrobacter sp. FB24; Salinispora tropica; Mesorhizobium sp. BNC1; Dickeya dadantii 3937; Magnetospirillum magneticum; Accumulibacter phosphatis; Rhodobacter sphaeroides KD131; Aromatoleum aromaticum EbN1; Arthrobacter aurescens; Arthrobacter chlorophenolicus; Pantoea vagans; Pseudomonas mendocina ymp; Opitutus terrae; Bacteroides fragilis YCH46; Alkaliphilus metalliredigens; Zunongwangia profunda; Kineococcus radiotolerans; Rhodopseudomonas palustris HaA2; Photorhabdus luminescens; Shewanella baltica OS195; Gordonia bronchialis; Desulfovibrio magneticus; Acidovorax avenae; Azorhizobium caulinodans; Pirellula staleyi; Xanthomonas campestris pv. vesicatoria; Herbaspirillum seropedicae; Lysinibacillus sphaericus; Candidatus Koribacter versatilis; Comamonas testosteroni; Ochrobactrum anthropi; Geodermatophilus obscurus; Bacteroides thetaiotaomicron; Rhodopseudomonas palustris BisA53; Shewanella woodyi ATCC 51908; Rhodopseudomonas palustris BisB18; Thermomonospora curvata; Nocardioides sp. JS614; Colwellia psychrerythraea; Shewanella piezotolerans WP3; Erwinia billingiae; Salinispora arenicola; Polaromonas naphthalenivorans; Mycobacterium abscessus ATCC 19977; Serratia proteamaculans; Desulfobacterium autotrophicum; Verminephrobacter eiseniae; Xanthomonas oryzae PXO99A; Bordetella bronchiseptica; Flavobacterium johnsoniae; Clostridium beijerinckii; Vibrio vulnificus YJ016; Bordetella petrii; Xanthobacter autotrophicus; Azotobacter vinelandii; Desulfitobacterium hafniense Y51; Paracoccus denitrificans; Ralstonia solanacearum GMI1000; Mycobacterium avium 104; Nakamurella multipartita; Rhodopseudomonas palustris TIE-1; Desulfatibacillum alkenivorans; Herpetosiphon aurantiacus; Cyanothece sp. ATCC 51142; Cyanothece sp. PCC 7425; Sphingomonas wittichii; Agrobacterium tumefaciens C58; Ralstonia pickettii 12D; Agrobacterium vitis S4; Pseudomonas putida GB-1; Caulobacter sp. K31; Mycobacterium marinum M; Polaromonas sp. JS666; Escherichia coli O157:H7 EC4115 (EHEC); Photobacterium profundum; Nocardiopsis dassonvillei; Enterobacter cloacae subsp. cloacae ATCC 13047; Mycobacterium gilvum; Bacillus megaterium QM B1551; Pseudomonas syringae pv. tomato DC3000; Cyanothece sp. PCC 7424; Dyadobacter fermentans; Pseudomonas fluorescens Pf0-1; Klebsiella pneumoniae 342; Burkholderia glumae; Bacillus anthracis CDC 684; Conexibacter woesei; Nocardia farcinica; Brevibacillus brevis; Mycobacterium sp. KMS; Mycobacterium vanbaalenii; Paenibacillus polymyxa SC2; Vibrio harveyi; Delftia acidovorans; Rhizobium etli CIAT 652; Anabaena sp. PCC7120; Pseudomonas fluorescens Pf-5; Methylobacterium extorquens AM1; Paenibacillus sp. JDR-2; Sinorhizobium medicae; Micromonospora aurantiaca; Geobacillus sp. Y412MC10; Burkholderia multivorans ATCC 17616 (JGI); Variovorax paradoxus S110; Pseudomonas aeruginosa PA7; Azospirillum sp. B510; Microcystis aeruginosa; Cupriavidus metallidurans; Stackebrandtia nassauensis; Pseudomonas fluorescens SBW25; Methylobacterium radiotolerans; Rhodococcus erythropolis; Ralstonia eutropha JMP134; Ralstonia eutropha H16; Cyanothece sp. PCC 7822; Agrobacterium radiobacter K84; Nostoc punctiforme; Methylobacterium sp. 4-46; Frankia alni; Mycobacterium smegmatis; Haliangium ochraceum; Hahella chejuensis; Achromobacter xylosoxidans; Burkholderia sp. CCGE1002; Actinosynnema mirum; Spirosoma linguale; Kribbella flavida; Frankia sp. EuI1c; Streptomyces griseus; Rhizobium leguminosarum; Burkholderia pseudomallei 1106a; Frankia sp. EAN1pec; Chitinophaga pinensis; Saccharopolyspora erythraea; Mesorhizobium loti; Myxococcus xanthus; Rhodopirellula baltica; Burkholderia phymatum; Burkholderia vietnamiensis; Bradyrhizobium sp. BTAi1; Streptomyces avermitilis; Candidatus Solibacter usitatus; Streptomyces coelicolor; Methylobacterium nodulans; Bradyrhizobium japonicum; Acaryochloris marina; Burkholderia xenovorans; Streptomyces scabiei; Catenulispora acidiphila; Streptosporangium roseum; Amycolatopsis mediterranei; Sorangium cellulosum*.
